# Supplementary material for: Harnessing mechanical instabilities at the nanoscale to achieve ultra-low stiffness metals
Source: Nat Commun. 2017 Oct 26;8:1137. doi: 10.1038/s41467-017-01260-6 (PMC5658392; doi:10.1038/s41467-017-01260-6)
Supplement: Supplementary file 1 — Supplementary Information [file 41467_2017_1260_MOESM1_ESM.pdf]

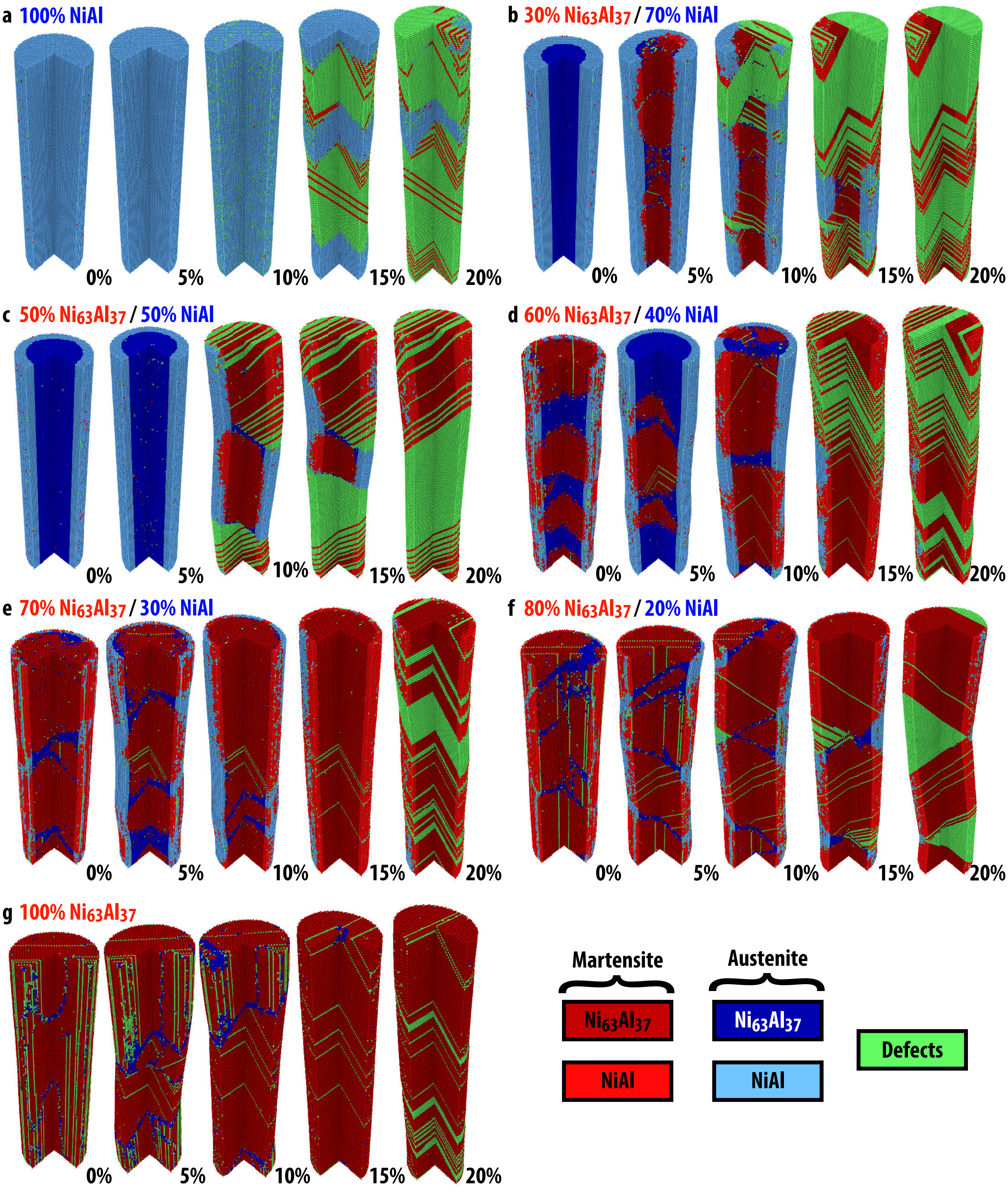

Supplementary Figure 1: Atomic structures in T-Core metamaterial nanowires. Representative structures for **(a)** homogeneous NiAl, **(b)** 30, **(c)** 50, **(d)** 60, **(e)** 70, and **(f)** 80% T-Core, and **(g)** homogeneous $Ni_{63}Al_{37}$ nanowires between 0 and 20% strain in 5% increments. Surface atoms and atoms in the front quarter were removed for clarity.

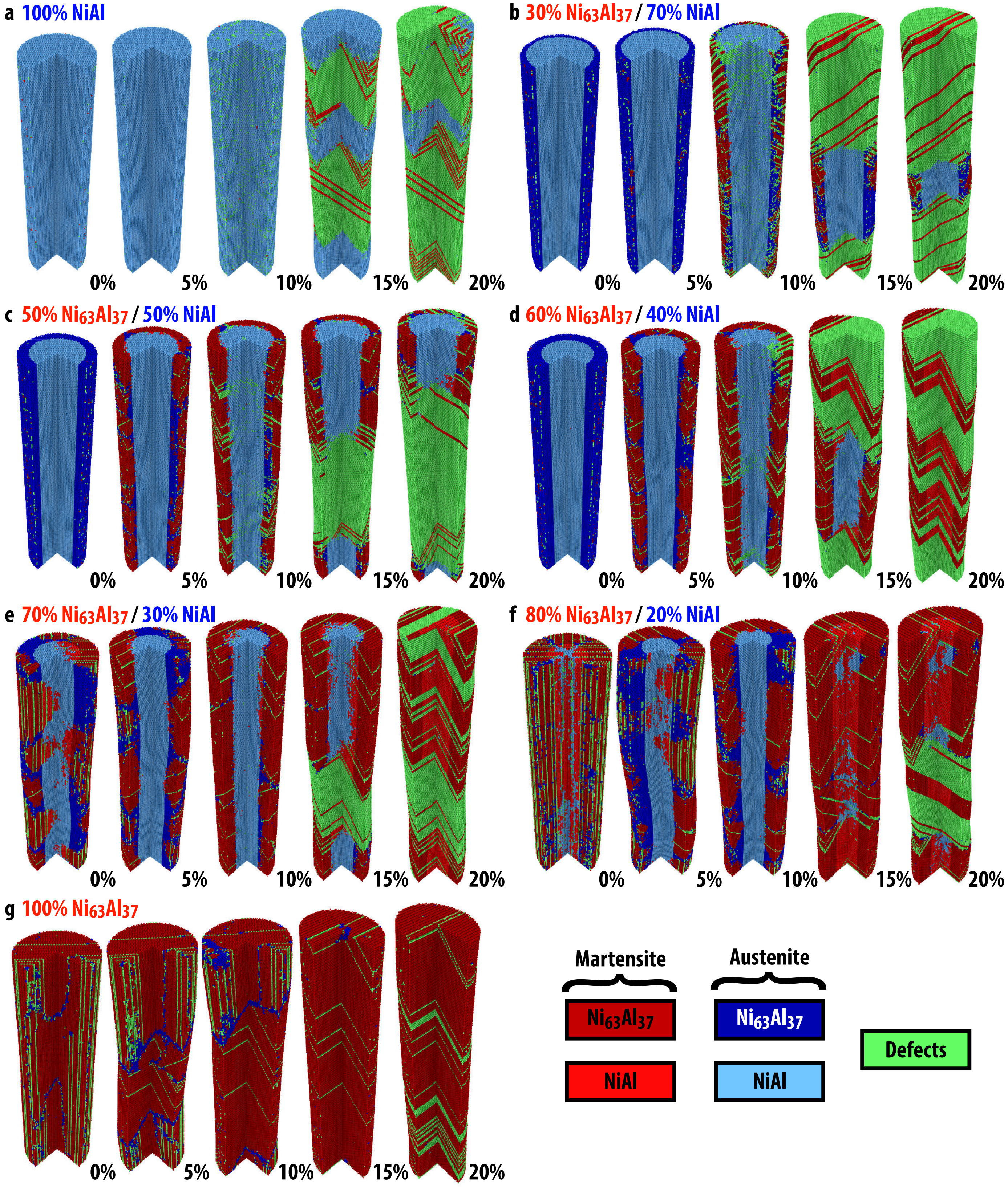

Supplementary Figure 2: Atomic structures in T-Shell metamaterial nanowires. Representative structures for **(a)** homogeneous NiAl, **(b)** 30, **(c)** 50, **(d)** 60, **(e)** 70, and **(f)** 80% T-Shell, and **(g)** homogeneous $Ni_{63}Al_{37}$ nanowires between 0 and 20% strain in 5% increments. Surface atoms and atoms in the front quarter were removed for clarity.

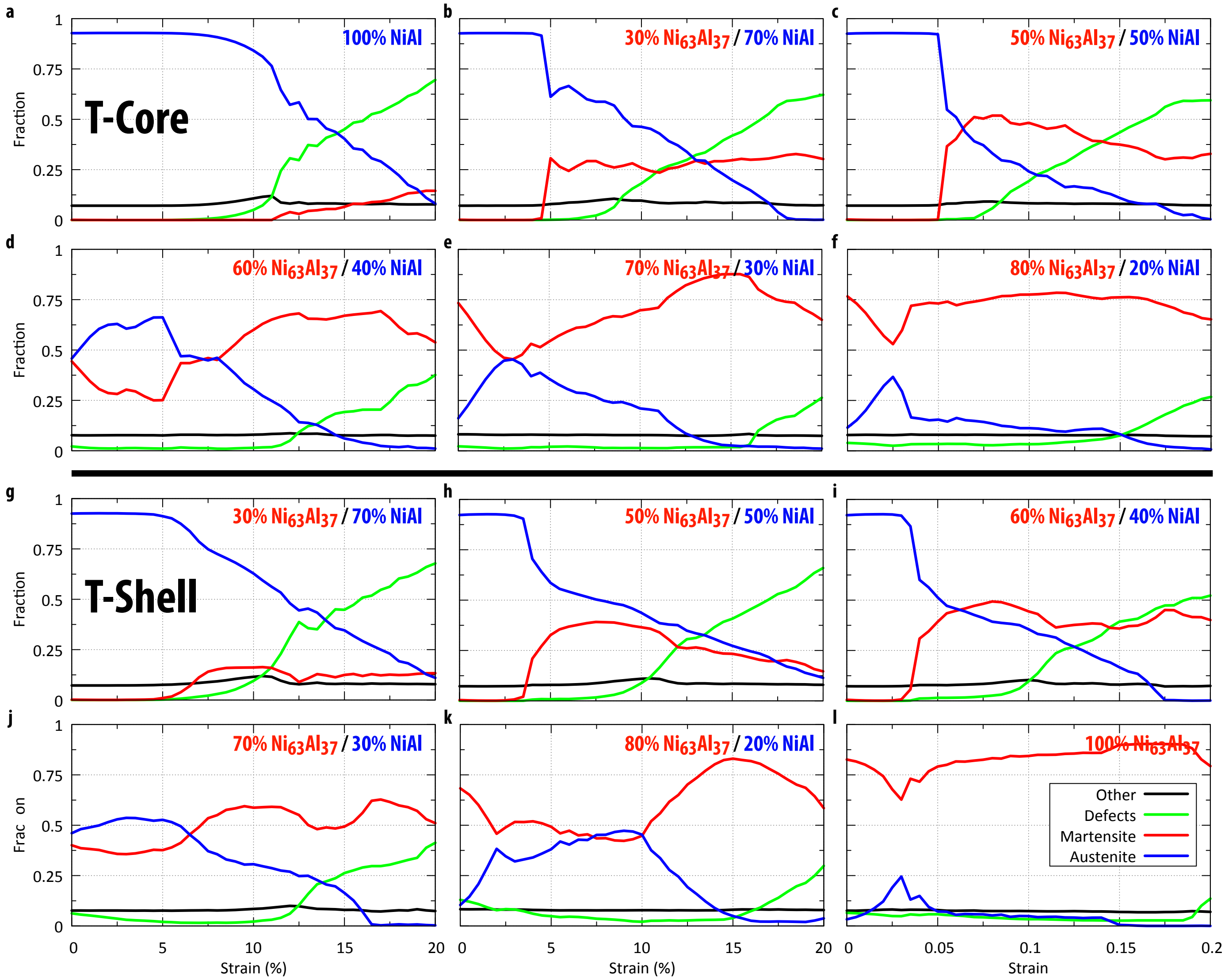

Supplementary Figure 3: Common neighbor analysis for metamaterial nanowires. **(a)** Homogeneous NiAl; **(b)** 30, **(c)** 50, **(d)** 60, **(e)** 70, and **(f)** 80% T-Core; **(g)** 30, **(h)** 50, **(i)** 60, **(j)** 70, and **(k)** 80% T-Shell; and **(l)** homogeneous $Ni_{63}Al_{37}$ nanowires between 0 and 20% strain for the same structures as Supplementary Figures 1-2. Defects (green) include stacking faults and highly strained unstable martensite phase.

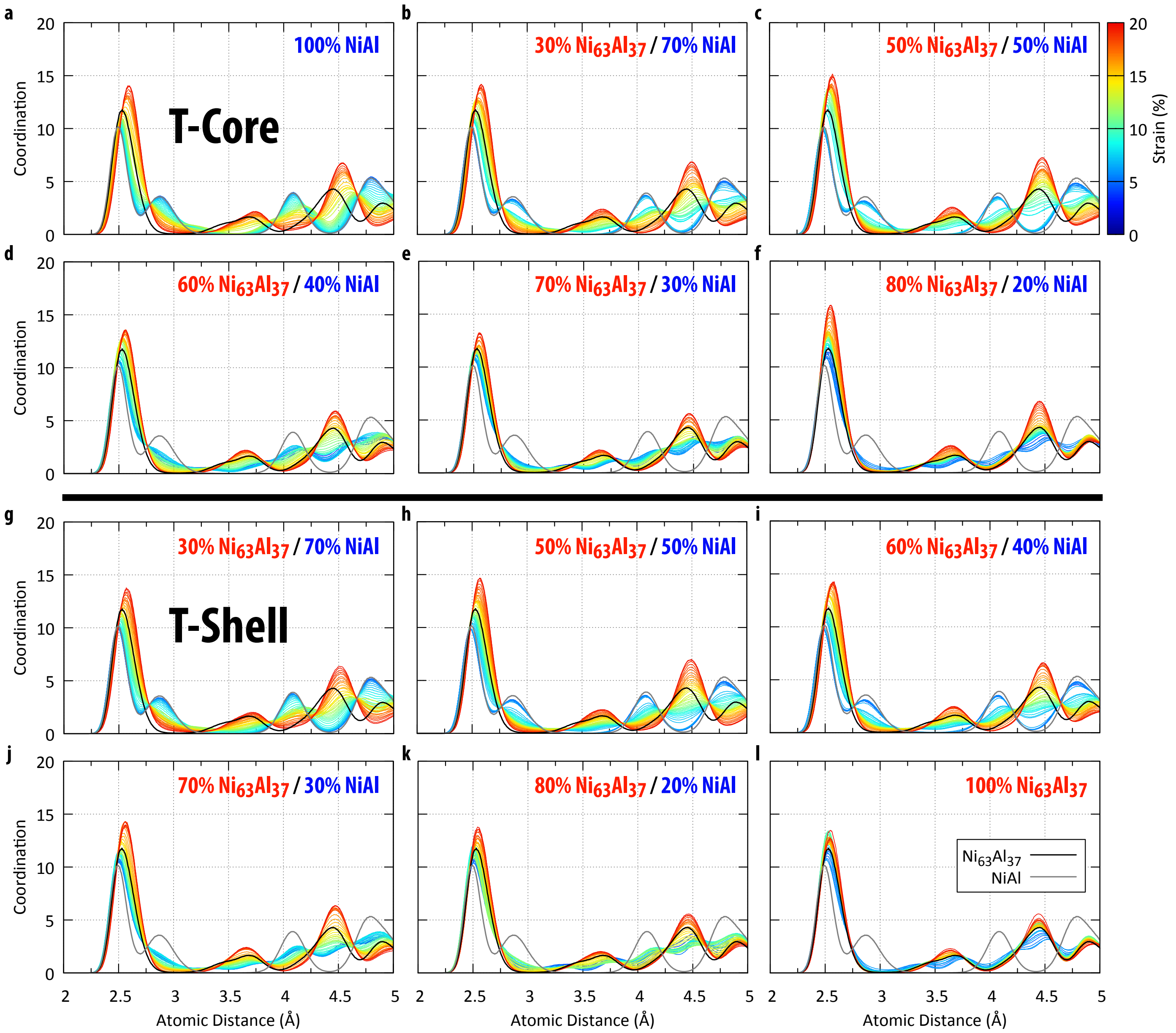

Supplementary Figure 4: Radial distribution functions for metamaterial nanowires. **(a)** Homogeneous NiAl;**(b)** 30, **(c)** 50, **(d)** 60, **(e)** 70, and **(f)** 80% T-Core; **(g)** 30, **(h)** 50, **(i)** 60, **(j)** 70, and **(k)** 80% T-Shell; and **(l)** homogeneous $Ni_{63}Al_{37}$ nanowires between 0 and 20% strain for the same structures as Supplementary Figures 1-2. Unstrained homogeneous $Ni_{63}Al_{37}$ and NiAl nanowires in black and grey, respectively.

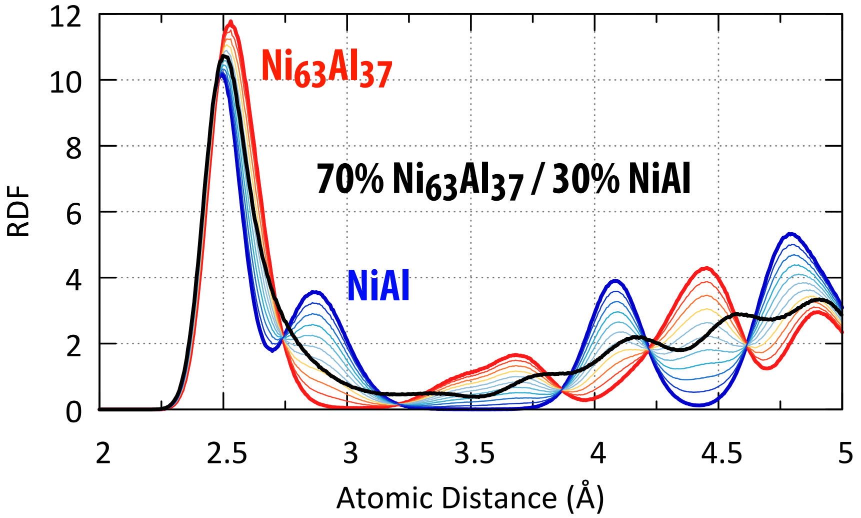

Supplementary Figure 5: Complex mixing within metatmaterial radial distribution function. Rule of mixtures applied to the RDF for homogeneous NiAl (blue) and $Ni_{63}Al_{37}$ (red) nanowires contrasted with the 70% T-Shell metamaterial nanowire RDF in black.

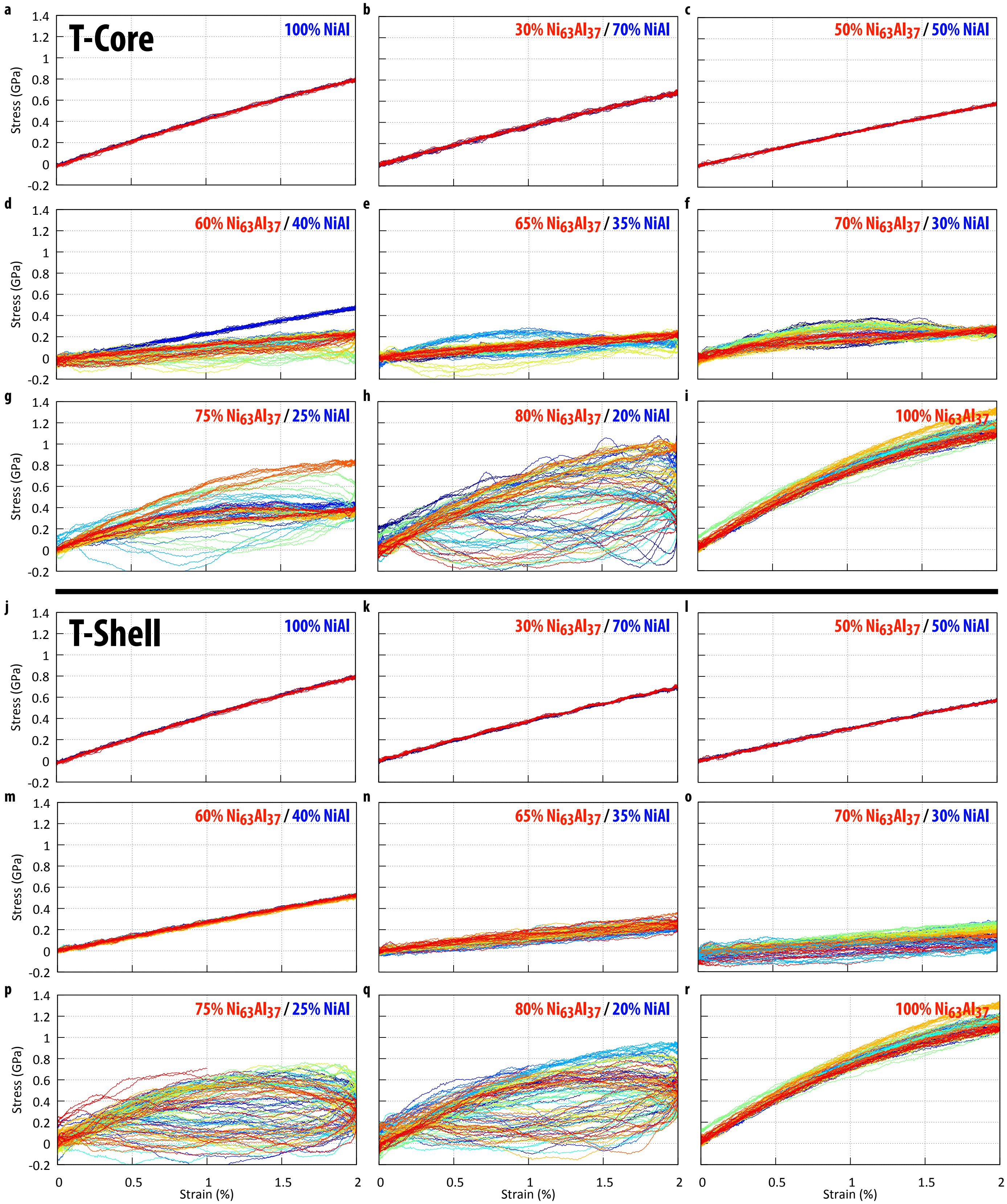

Supplementary Figure 6: Elastic cycling of metamaterial nanowires. **(a)** Homogeneous NiAl, **(b)** 30, **(c)** 50, **(d)** 60, **(e)** 65, **(f)** 70, **(g)** 75, and **(h)** 80% T-Core, and **(i)** homogeneous $Ni_{63}Al_{37}$ and **(j)** - **(r)** matching composition T-Shell nanowires for five cycles between 0 and 2% strain. Each color refers to a unique sample.

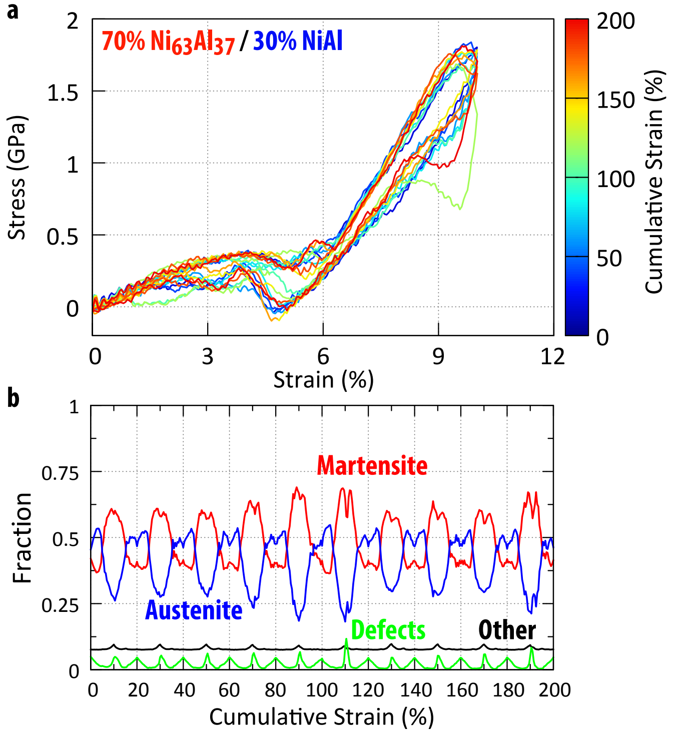

Supplementary Figure 7: Plastic cycling of a metamaterial nanowire. 70% T-Shell nanowire **(a)** stress-strain response and **(b)** common neighbor analysis for ten cycles between 0 and 10% strain.

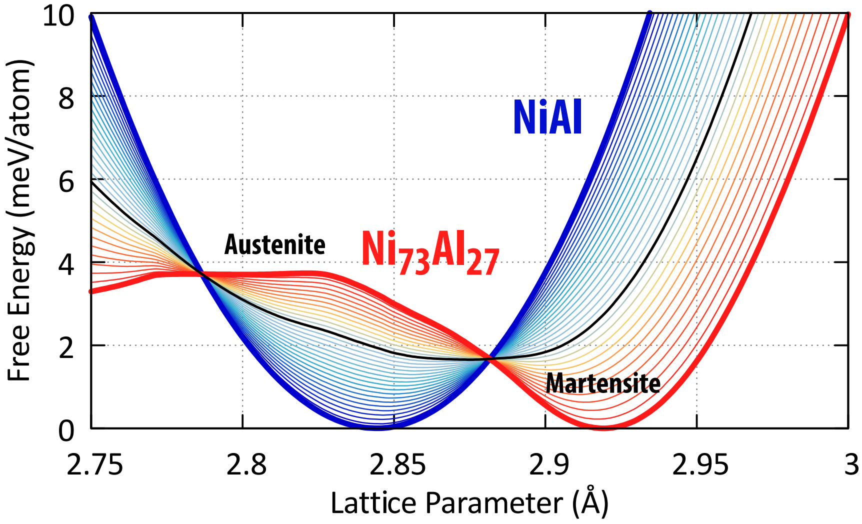

Supplementary Figure 8: Purja-Pun potential free energy landscape. Free energy as a function of in-plane lattice parameter calculated by MD simulations with the Purja-Pun potential of bulk $Ni_{73}Al_{27}$ (thick red) and NiAl (thick blue) and interpolated with rule of mixtures. The combination of 60 at. % $Ni_{73}Al_{37}$ and 40 at. % NiAl is highlighted in black. The martensite and austenite are labeled for $Ni_{73}Al_{37}$.

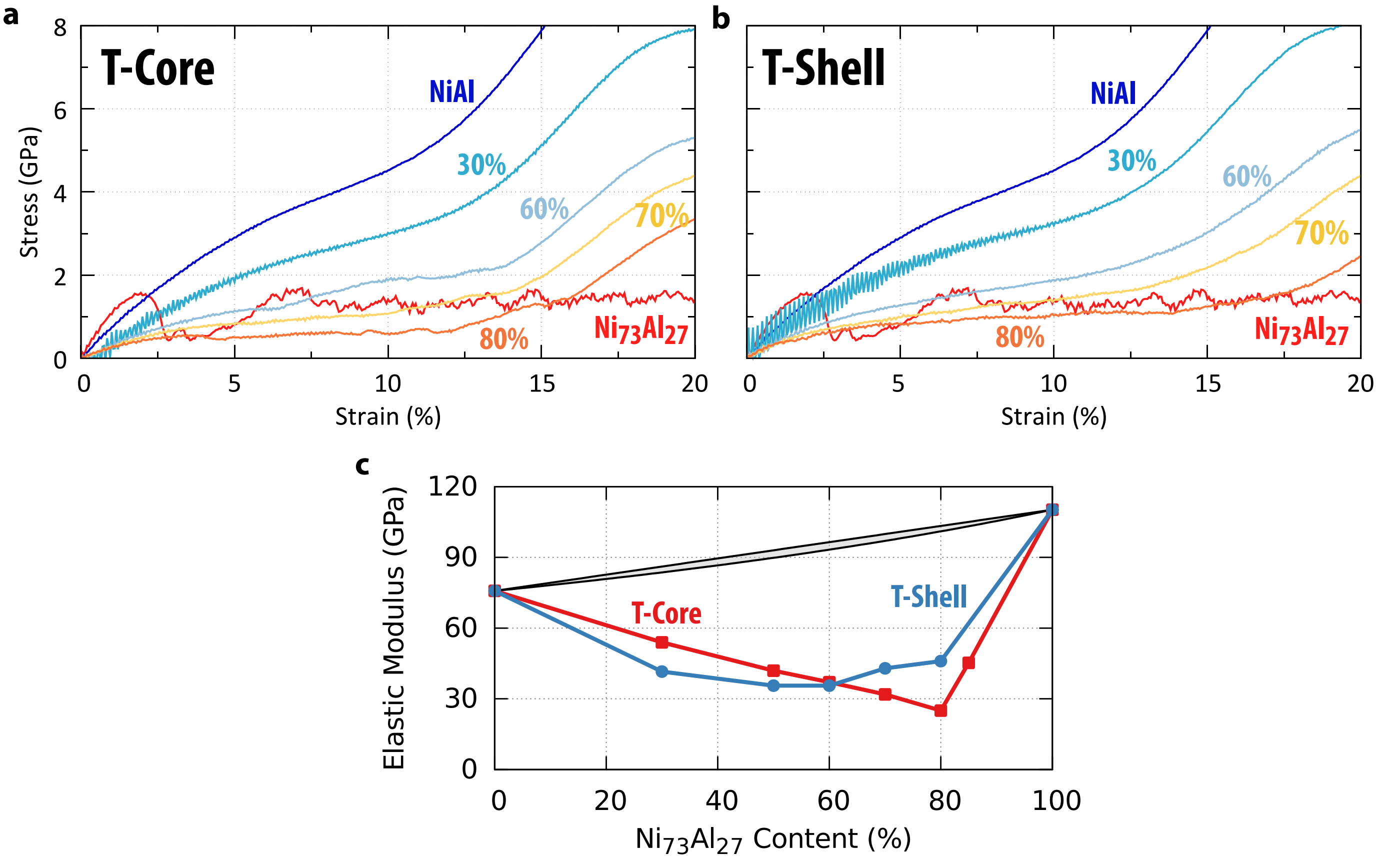

Supplementary Figure 9: Purja-Pun potential nanowire mechanical response. Stress-strain curves for representative metamaterial nanowires with the Purja-Pun potential for **(a)** T-Core and **(b)** T-Shell configurations where homogeneous nanowires are labeled as NiAl and $Ni_{73}Al_{27}$ and metamaterial nanowires are identified by composite fraction of the $Ni_{73}Al_{27}$ phase. **(c)** Resulting stiffness as a function of $Ni_{73}Al_{27}$ composite fraction with black lines (and grey shading) showing standard rule of mixtures.

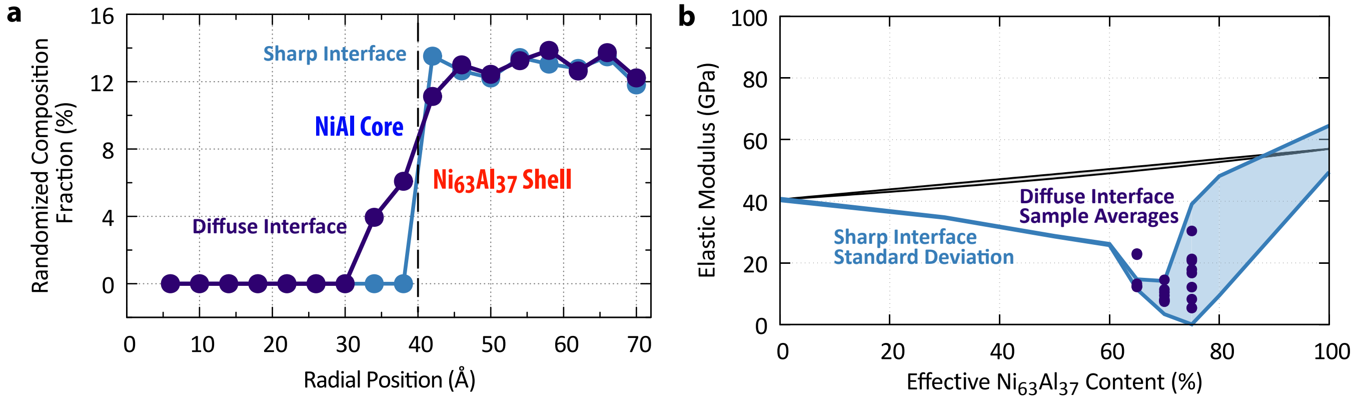

Supplementary Figure 10: Diffusive interface metamaterial nanowires. Comparison of **(a)** radial composition profiles and **(b)** elastic cycling response for diffuse and sharp interface nanowires. Standard deviations from Fig. 5 (Supplementary Fig. 6) sample averages.

Supplementary Table 1: Crystal structures for bulk NiAl and $Ni_{63}Al_{37}$

	Lattice parameter (Å)			Non-orthogonal Lattice angles (°)
	a	b	c	γ
NiAl bulk (B2 austenite)	2.90	2.90	2.90	-
$Ni_{63}Al_{37}$ bulk (monoclinic martensite)	4.16	4.34	2.62	88.4
Highly-strained $Ni_{63}Al_{37}$ bulk at 15% strain (tetragonal martensite)	3.74	2.57	2.58	-

Supplementary Note 1: Additional nanowire structural results

Complementary results to the main paper are shown for metamaterial nanowires (details in Methods) with $Ni_{63}Al_{37}$ fractions 30, 50, 60, 70, and 80 at. % for both T-Core and T-Shell. For reference, corresponding results for the NiAl and $Ni_{63}Al_{37}$ wires are also shown. Atomistic structures are shown in Supplementary Figures 1 and 2 for T-Core and T-Shell configurations, respectively. Similarly, Supplementary Figures 3 and 4 shows CNA and RDF results, respectively, for all cases. In the lowest $Ni_{63}Al_{37}$ fractions, the transformations to martensite are abrupt and limited only to the $Ni_{63}Al_{37}$ region of the wire (T-Shell or T-Core); values exhibiting ultra-low stiffness instead contain a balance of both austenite and martensite in both regions with smoother transformation to higher martensite fraction. All metamaterial cases show some degree of softening, with corresponding degrees of phase coexistence and variation in local lattice parameter.

Supplementary Note 2: Nanowire cyclic mechanical testing

Cyclic loading was performed on the nanowires (described in Methods) by repeatedly extending and compressing the wires. Supplementary Figure 6 shows the reproducibility of the stress-strain results (for both T-Core and T-Shell). Both configurations show similar trends: little variability, lack of hysteresis, and some softening in low fractions; high variability and high hysteresis at high fractions, with both high and low stiffness samples; and lowest stiffness, consistency, and very little hysteresis between 60 and 70 at. % $Ni_{63}Al_{37}$. High variability in the high composite fraction cases comes from the wide range of possible variant and phase combinations; high hysteresis occurs for the systems transforming between single-phase (martensite) and multi-phase states, unlike the lowest stiffness cases with a continual balance of martensite and austenite.

Cycling with full transformation (but prior to the unstable martensite variant) was also tested for one 70 at. % T-Shell sample at 5×10^{8} s^{-1}, between 0 and 10% strain for ten cycles with a linear (triangular) strain profile. The stress-strain curves and CNA results in Supplementary Figure 7 further highlight the fully reversible nature of the transformations and their reproducibility. The cycles show consistent low stiffness behavior and excellent reversibility, even with occasional deviation from the average loop behavior, which are erased by the following cycle. Hysteresis in the cycle occurs due to full transformation to martensite from the initially multi-phase system.

Supplementary Note 3: Nanolaminate mechanical testing

Nanolaminate systems were tested in order to compare to the nanowires results; see Supplementary Methods 2 for simulation details. Nanolaminate stiffness as a function of $Ni_{63}Al_{37}$ fraction is shown in the inset to Fig. 5. Stiffness values were taken from single sample stress-strain results; variability is expected to be similar to the nanowire results. The stiffness trend is very similar to the nanowires, but with shifts in minimum stiffness with increasing geometric constraint: 70-75 at. % T-Shell, 60-65 at. % T-Core, and 45 at. % for laminates. The smaller stiffness reductions can be explained by the constraints of surrounding material reducing both shared phase stability and multiple martensitic domain formation. Softening in the laminate structures therefore comes from the more general increased range of lattice parameters with comparable free energies.

Supplementary Note 4: Low-stiffness nanowires using Purja Pun potential

In order to demonstrate that the low stiffness results are not specific to the interatomic potential used, we show results with a Ni-Al potential developed by Purja Pun et al[4]. This second potential was fitted to experimental lattice parameters, formation energies, and elastic constants of B2 NiAl and density functional theory formation energies for numerous other ordered phases[5]. It was shown to capture martensitic behavior only under relatively large hydrostatic stress or in the presence of defects (e.g. free surfaces)[6].

As with the Farkas potential, biaxial strain tests (see Methods) were performed to predict the ideal compositions and core/shell fractions for stiffness modification, shown in Supplementary Figure 9. This potential does not predict martensitic transformation for $Ni_{63}Al_{37}$ nanowires at 300 K and 1 atm; therefore, instead, $Ni_{73}Al_{27}$ was used as the transforming phase. Even with this increase, the model does not produce ideal overlap between NiAl and $Ni_{73}Al_{27}$.

Mechanical testing (see details in Supplementary Methods 3) was performed on comparable metamaterial fractions as the main results and for homogeneous NiAl and $Ni_{73}Al_{27}$ nanowires. Stress-strain results and trends for stiffness are shown in Supplementary Figure 10, taken from single sample stress-strain curves; variability is expected to be similar to the main (Farkas potential) results. For the Purja Pun potential, the lowest stiffness came with the 80 at. % $Ni_{73}Al_{37}$ in the T-Core configuration, owing to the limited driving force of martensite formation at 300 K with this potential and the corresponding lack of phase coexistence and single martensitic domain formation. Most importantly, this result matches the predictions from the energy landscapes; the landscape overlap for this material is less than ideal and the stiffness engineering reflects this. Therefore, this entirely separate interatomic model validates the main idea of utilizing mechanical instabilities by epitaxial integration and landscape engineering.

Supplementary Note 5: Diffuse interface nanowires

To establish that the ultra-low stiffness results are insensitive to diffusion between components, we created 60-70 at. % T-Shell nanowires with a diffuse compositional interface. The shell region was built as described in the Methods; within a distance of three unit cells radially nearest the interface, the target composition was a linear interpolation between 0 and 13% added (randomized) Ni. Supplementary Figure 8a compares the fraction of randomized nickel as a function of nanowire radius. The example from the main paper shows the abrupt change from the NiAl to $Ni_{63}Al_{37}$, contrasted by the diffuse systems. For all structures there is noise in the randomized section on a per layer basis, though the overall composition in the core and shell regions average to the prescribed fraction. Due to the added randomized composition for the graded interfaces, each is compared to T-Shell sharp interface systems with 5% larger shell regions. The elastic response is shown in Supplementary Figure 8b. When comparing the correct phase fraction samples, the results for ten samples with independently randomized composition for each T-Shell fraction fall almost entirely within the ranges of the sharp interface samples from the main paper. For a minority of cases of graded interface 65 (effective) at. % T-Shell nanowires fall outside the sharp interface range as the specific distribution of randomized nickel prefers a single phase structure, as in the sharp interface 60 at. % T-Shell.

Supplementary Methods

1. Phase characterization

Atomic structure characterization through radial distribution functions (RDF) was performed within LAMMPS. Common neighbor analysis (CNA) was also used to characterize the structures throughout the simulations using the adaptive algorithm[1] within OVITO. This method loops over all atoms and for each examines the *n* relevant nearest neighbors to compare to desired crystal structure "signatures" (FCC, BCC, and HCP). Notably, a separate cutoff value is used for each atom and potential structure to determine bonds. An atom is defined to be of a given structure by the number of bonds of varying type, where each bond is defined by number of neighbors, number of common neighbors that share a bond, and the maximum chain that connects common neighbor bonds. For example, FCC atoms have 12 bonds, where each pair of neighbors share four common neighbors; those common neighbors are connected by two bonds, with a maximum chain length between those two bonds of one. Atoms characterized as BCC are reported as austenite, HCP as martensite, and FCC as defects, including stacking faults and the unstable martensite at strains above 10%. The atoms not matching the signatures of any of the aforementioned structures are classified as "Other". Surface atoms (all "Other") are removed from all snapshots for clarity; the remaining "Other" atoms are included with Defects in snapshots. Within CNA plots, all "Other" atoms (including surface) are shown separately.

Supplementary Table 1 describes the crystal structures for the austenite, martensite, and highly-strained martensite. Lattice parameters and angles were averaged over at least 1000 random samples within the structures. The NiAl bulk austenite is a standard B2 cubic structure. The martensite is the stable phase for the $Ni_{63}Al_{37}$ and is monoclinic with two directions that expand, one direction that contracts, and one angle that deviates from 90° during transformation. In contrast, the unstable, strained martensite which forms in all structures tested at large strains (above 10%) is tetragonal, with two short directions and one long. The values listed in Supplementary Table 1 for the strained martensite is one example, the $Ni_{63}Al_{37}$ phase at 15% strain, after the entire sample has transformed. The martensitic phases were characterized as in Morrison et al[2]. Each metamaterial nanowire and laminate contains these phases, distorted commensurate with the landscape engineering.

2. Nanolaminate simulation details

Epitaxial nanolaminates of one period of each $Ni_{63}Al_{37}$ and NiAl were created as cubes with 23.2 nm side lengths, built with $\{100\}$ orientation, with periodic boundary conditions in all directions, consisting of 1,024,000 atoms, and with comparable composite fractions to the nanowire results. Equilibration was first performed with all directions and angles relaxed independently. Laminates with $Ni_{63}Al_{37}$ fractions less than or equal to 50 at. % were equilibrated at 300 K with 1 atm along each direction and 0 atm allowing each angle to relax with an NPT ensemble. For fractions of $Ni_{63}Al_{37}$ greater than 50 at. %, the laminate structures initialized in the B2 phase showed low stiffness as they strongly preferred to transform to martensite almost immediately after loading. Softening prior to martensitic transformation is well known[3] and this mechanism is distinct from the purposeful landscape engineering of this paper. For these cases, the structures were cooled from 300 K to 25 K through the martensite start (M_S) transformation temperature, heated back to 300 K, and then equilibrated in order to begin the uniaxial deformation with martensitic structures. All equilibration was run for 100 ps. The structures were then deformed identically to the nanowires (deformation to 20% engineering strain in 400 ps for a strain rate of $5x10^8$ s^{-1} under an NVT

ensemble), with deformation parallel to the laminate direction and all directions and angles not strain controlled allowed to relax with the same pressure constraints as equilibration.

3. Nanowires using Purja Pun potential simulation details

The nanowires using the second interatomic potential initially measured 11.4 nm in diameter and 34.5 nm long and contained 302,520 atoms, built with a $\{100\}$ orientation, and a periodic boundary only along the wire axis. Each wire was thermalized and strained as in the main paper (see Methods).

Supplementary References

1. Stukowski, A. Structure identification methods for atomistic simulations of crystalline materials. *Model. Simul. Mater. Sci. Eng.* **20,** 45021 (2012).
2. Morrison, K. R., Cherukara, M. J., Guda Vishnu, K. & Strachan, A. Role of atomic variability and mechanical constraints on the martensitic phase transformation of a model disordered shape memory alloy via molecular dynamics. *Acta Mater.* **69,** 30–36 (2014).
3. Ding, X., Suzuki, T., Ren, X., Sun, J. & Otsuka, K. Precursors to stress-induced martensitic transformations and associated superelasticity: Molecular dynamics simulations and an analytical theory. *Phys. Rev. B - Condens. Matter Mater. Phys.* **74,** 23–27 (2006).
4. Purja Pun, G. P. & Mishin, Y. Development of an interatomic potential for the Ni-Al system. *Philos. Mag.* **89,** 3245–3267 (2009).
5. Mishin, Y., Mehl, M. J. & Papaconstantopoulos, D. A. Embedded-atom potential for B2−NiAl. *Phys. Rev. B* **65,** 224114 (2002).
6. Purja Pun, G. P. & Mishin, Y. Molecular dynamics simulation of the martensitic phase transformation in NiAl alloys. *J. Phys. Condens. Matter* **22,** 395403 (2010).
