# Supplementary material for: Harnessing mechanical instabilities at the nanoscale to achieve ultra-low stiffness metals
Source: Nat Commun. 2017 Oct 26;8:1137. doi: 10.1038/s41467-017-01260-6 (PMC5658392; doi:10.1038/s41467-017-01260-6)
Supplement: Supplementary file 3 — Description of Additional Supplementary Files [file 41467_2017_1260_MOESM3_ESM.pdf]

Description of Additional Supplementary Files

File Name: Supplementary Movie 1
Description: Deformation to 20% strain for the homogeneous Ni63Al37 nanowire (Fig. 3a).

File Name: Supplementary Movie 2
Description: Deformation to 20% strain for the 70 at. % T-Shell nanowire (Fig. 3b).

File Name: Supplementary Movie 3
Description: Cyclic loading (up to 2% strain) for the minimum stiffness 70 at. % TShell nanowire, approximately 4 GPa (Supplementary Fig. 6o)

File Name: Supplementary Movie 4
Description: Cyclic loading (up to 2% strain) for an average stiffness 70 at. % TShell nanowire, approximately 8 GPa (Supplementary Fig. 6o)

File Name: Supplementary Movie 5
Description: Cyclic loading (up to 2% strain) for the minimum stiffness 60 at. % TCore nanowire, approximately 2 GPa (Supplementary Fig. 6d)

File Name: Supplementary Movie 6
Description: Cyclic loading (up to 2% strain) for an average stiffness 65 at. % TCore nanowire, approximately 10 GPa (Supplementary Fig. 6e)
